# Supplementary material for: Obesity as a Risk Factor for Venous Thromboembolism Recurrence: A Systematic Review
Source: Medicina (Kaunas). 2022 Sep 16;58(9):1290. doi: 10.3390/medicina58091290 (PMC9503246; doi:10.3390/medicina58091290)
Supplement: Supplementary file 1 [file medicina-58-01290-s001.zip › Supplementary Tables S2, S3 and S4.pdf]

Table S2. The Newcastle-Ottawa scale [1] for quality assessment of cohort and case controls studies

STUDY [Ref.]	SELECTION (max 4 stars)	COMPARABILITY (max 2 stars)	OUTCOME (max 3 stars)	OVERALL QUALITY SCORE (max 9 stars)
García-Fuster et al [2]	***	**	***	8
Rodger et al [3]	****	**	***	9
Eichinger et al [4]	****	**	**	8
Di Nisio et al [5]	***	**	**	7
Farzamnia et al [6]	****	*	*	6
Olié et al [7]	****	**	***	9
Rodger et al [8]	****	**	**	8
Franco Moreno et al [9]	****	**	**	8
Huang et al [10]	****	*	**	7
Asim et al [11]	****	**	*	7
Vučković et al [12]	****	**	**	8
Mueller et al [13]	***	**	***	8
Stewart et al [14]	****	*	**	7
Stewart et al [15]	***	**	**	7
Giorgi-Pierfranceschi et al [16]	***	**	*	6
Cardinal et al [17]	***	*	*	5
Weitz et al [18]	****	*	***	8

Table S3. Risk of bias of randomized controlled trials included in the analysis performed with the Cochrane collaboration risk of bias tool (CCRBT) for the randomized controlled trial [19]

Reference	Randomization process	Deviations from intended interventions	Missing outcome data	Measurement of the outcome	Selection of the reported result	Overall risk-of-bias judgement
Di Nisio et al [20]	Low risk	Low risk	Low risk	Low risk	Low risk	Low risk
Beemen et al [21]	Low risk	Low risk	Low risk	Low risk	Low risk	Low risk

Table S4. Quality assessment of cross-sectional studies performed with AXIS scale [22]. Abbreviations, NS; Not specified,

Reference	Linnemann et al [23]
**Introduction**	
1. Were the aims/objectives of the study clear?	Yes
**Methods**	
2 Was the study design appropriate for the stated aim(s)?	Yes
3 Was the sample size justified?	No
4 Was the target/reference population clearly defined? (Is it clear who the research was about?)	Yes
5 Was the sample frame taken from an appropriate population base so that it closely represented the target/reference population under investigation?	NS
6 Was the selection process likely to select subjects/participants that were representative of the target/reference population under investigation?	Yes
7 Were measures undertaken to address and categorize non-responders?	NS
8 Were the risk factor and outcome variables measured appropriate to the aims of the study?	Yes

9 Were the risk factor and outcome variables measured correctly using instruments/ measurements that had been trialled, piloted or published previously?	Yes
10 Is it clear what was used to determined statistical significance and/or precision estimates? (e.g., p values, CIs)	Yes
11 Were the methods (including statistical methods) sufficiently described to enable them to be repeated?	Yes
**Results**	
12 Were the basic data adequately described?	Yes
13 Does the response rate raise concerns about nonresponse bias?	No
14 If appropriate, was information about nonresponders described?	NS
15 Were the results internally consistent?	NS
16 Were the results for the analyses described in the methods, presented?	Yes
**Discussion**	
17 Were the authors' discussions and conclusions justified by the results?	Yes
18 Were the limitations of the study discussed?	Yes
Others	

19 Were there any funding sources or conflicts of interest that may affect the authors' interpretation of the results?	NS
20 Was ethical approval or consent of participants attained?	Yes

REFERENCES

1. Wells G.A., Shea B., O'Connell D., Peterson J., Welch V., Losos M., et al. The Newcastle–Ottawa scale (NOS) for assessing the quality of nonrandomised studies in meta-analyses. Available from: http://www.ohri.ca/programs/clinical_epidemiology/oxford.asp [Accessed 4th April 2022].
2. García-Fuster M.J., Forner M.J., Fernández C., Gil J., Vaya A., Maldonado L. Long-term prospective study of recurrent venous thromboembolism in patients younger than 50 years. Pathophysiol Haemost Thromb 2005, 34, 6-12.
3. Rodger M.A., Kahn S.R., Wells P.S., Anderson D.A., Chagnon I., Le Gal G., et al. Identifying unprovoked thromboembolism patients at low risk for recurrence who can discontinue anticoagulant therapy. CMAJ 2008, 179, 417-26.
4. Eichinger S., Hron G., Bialonczyk C., Hirschl M., Minar E., Wagner O., et al. Overweight, obesity, and the risk of recurrent venous thromboembolism. Arch Intern Med 200,168, 1678-83.
5. Di Nisio M., Di Iorio A., Porreca E., Abate M., Ferrante N., Bandinelli S., et al. Obesity, poor muscle strength, and venous thromboembolism in older persons: the InCHIANTI study. J Gerontol A Biol Sci Med Sci 2011, 66, 320-5.
6. Farzamnia H., Rabiei K., Sadeghi M., Roghani F. The predictive factors of recurrent deep vein thrombosis. ARYA Atheroscler 2011, 7, 123-8.
7. Olié V., Zhu T., Martinez I., Scarabin P.Y., Emmerich J. Sex-specific risk factors for recurrent venous thromboembolism. Thromb Res 2012, 130, 16-20.
8. Rodger M.A., Scarvelis D., Kahn S.R., Wells P.S., Anderson D.A., Chagnon I., et al. Long-term risk of venous thrombosis after stopping anticoagulants for a first unprovoked event: A multi-national cohort. Thromb Res 2016, 143, 152-8.
9. Franco Moreno A.I., García Navarro M.J., Ortiz Sánchez J., Martín Díaz R.M., Madroñal Cerezo E., de Ancos Aracil C.L., et al. A risk score for prediction of recurrence in patients with unprovoked venous thromboembolism (DAMOVES). Eur J Intern Med 2016, 29, 59-64.
10. Huang W., Goldberg R.J., Anderson F.A., Cohen A.T., Spencer F.A. Occurrence and predictors of recurrence after a first episode of acute venous thromboembolism: population-based Worcester Venous Thromboembolism Study. J Thromb Thrombolysis 2016, 41, 525-38.
11. Asim M., Al-Thani H., El-Menyar A. Recurrent Deep Vein Thrombosis After the First Venous Thromboembolism Event: A Single-Institution Experience. Med Sci Monit 2017, 23, 2391-2399.
12. Vučković B.A., Cannegieter S.C., van Hylckama Vlieg A., Rosendaal F.R., Lijfering W.M. Recurrent venous thrombosis related to overweight and obesity: results from the MEGA follow-up study. J Thromb Haemost 2017, 15, 1430-1435.
13. Mueller C., Limacher A., Méan M., Rodondi N., Aujesky D. Obesity is not associated with recurrent venous thromboembolism in elderly patients: Results from the prospective SWITCO65+ cohort study. PLoS One 2017, 15; 12, e0184868.
14. Stewart, L.K., J.A. Kline. Metabolic Syndrome Increases Risk of Venous Thromboembolism Recurrence after Acute Pulmonary Embolism. Ann Am Thorac Soc, 2020, 17, 821-828.
15. Stewart L.K., Kline J.A. Metabolic syndrome increases risk of venous thromboembolism recurrence after acute deep vein thrombosis. Blood Adv 2020,4, 127-135.
16. Giorgi-Pierfranceschi M., López-Núñez J.J., Monreal M., Cattabiani C., Lodigiani C., Di Micco P., et al. Morbid Obesity and Mortality in Patients With VTE: Findings from Real-Life Clinical Practice. Chest 2020, 157, 1617-1625.
17. Cardinal R.M., D'Amico F., D'Addezio A., Dakers K., Castelli G. Safety and efficacy of direct oral anticoagulants across body mass index groups in patients with venous thromboembolism: a retrospective cohort design. J Thromb Thrombolysis 2021,52, 567-576.
18. Weitz J.I., Farjat A.E., Ageno W., Turpie A.G.G., Haas S., Goto S., et al. Influence of body mass index on clinical outcomes in venous thromboembolism: Insights from GARFIELD-VTE. J Thromb Haemost 2021,19, 3031-3043.
19. Higgins J.P.T., Altman D.G., Gøtzsche P.C., Jüni P., Moher D., Oxman A.D., et al. The Cochrane Collaboration's tool for assessing risk of bias in randomised trials. BMJ 2011;343.
20. Di Nisio M., Vedovati M.C., Riera-Mestre A., Prins M.H., Mueller K., Cohen A.T., et al. Treatment of venous thromboembolism with rivaroxaban in relation to body weight. A sub-analysis of the EINSTEIN DVT/PE studies. Thromb Haemost 2016, 116, 739-46.
21. Beenen L.F.M., Scheres L.J.J., Stoker J., Middeldorp S. Prognostic characteristics and body mass index in patients with pulmonary embolism: does size matter? ERJ Open Res 2020,6,00163-2019.
22. Downess M.J., Brennan M.L., Williams H.C., Dean R.S. Development of a critical appraisal tool to assess the quality of cross-sectional studies (AXIS). BMJ Open 2016,6e011458.
23. Linnemann B., Zgouras D., Schindewolf M., Schwonberg J., Jarosch-Preusche M., Lindhoff-Last E. Impact of sex and traditional cardiovascular risk factors on the risk of recurrent venous thromboembolism: results from the German MAISTHRO Registry. Blood Coagul Fibrinolysis 2008, 19, 159-65.
